# Evaluating Multimodal Vision Frontier Models for Grading Neuroforaminal Stenosis on Lumbar Spine MRIs

**DOI:** 10.1177/21925682261508040

**Published:** 2026-11-11

**Authors:** Ayesha Akbar Waheed, Ahmet Kartal, Sohail R. Daulat, Noel F. Manalil, Chiungwen D. Cheng, Ibrahim Hussain, Roger Härtl, Galal A. Elsayed, Harry Gebhard

**Affiliations:** 1 Department of Neurosurgery, University of Pittsburgh School of Medicine, Pittsburgh, PA, USA; 2 Department of Neurological Surgery, Och Spine at NewYork-Presbyterian Hospital, 12295 Weill Cornell Medicine, New York, NY, USA; 3 12216 Department of Neurosurgery, University of Arizona College of Medicine–Tucson, Tucson, AZ, USA; 4 Department of Neurosurgery, 695469 Medical University Lausitz – Carl Thiem, Cottbus, Germany

**Keywords:** Multimodal Vision Frontier Models (M-VFMs), Lumbar Spine MRI, Neuroforaminal Stenosis, Diagnostic Accuracy

## Abstract

**Study Design:**

Diagnostic accuracy study.

**Objective:**

To determine whether a general-purpose multimodal artificial intelligence (AI) model, Gemini 3 Pro, grades lumbar neuroforaminal stenosis on magnetic resonance imaging (MRI) in agreement with expert radiologists, particularly for severe stenosis, and whether a vertebral-level localizer overlay helps.

**Methods:**

One hundred lumbar MRIs from the Radiological Society of North America (RSNA) 2024 Lumbar Spine Degenerative Classification Dataset, graded by expert radiologists, provided 1,000 foramina (five levels, both sides) labeled normal/mild, moderate, or severe. Sagittal and axial T2-weighted series were converted into short scrolling videos and submitted to the model with a structured grading prompt; no task-specific training was performed. The primary outcome was agreement with expert grades (linearly weighted Cohen’s kappa, κw); secondary outcomes were accuracy, severe-stenosis sensitivity, direction of errors, and the localizer effect.

**Results:**

Agreement with expert grading was poor (κw 0.20, 95% confidence interval [CI] 0.14–0.26). Accuracy was 70.6% (95% CI 67.7–73.4) but reflected the 70.4% of foramina that were normal/mild. The model identified 17.9% of severe foramina (95% CI 8.9–30.4) and called 58.9% normal/mild. Errors were systematically toward undergrading (p < 0.001). The localizer did not improve accuracy (70.0% vs 70.6%, p = 0.590) and reduced severe sensitivity (3.6% vs 17.9%, p = 0.043).

**Conclusions:**

Without task-specific training, a general-purpose AI model reached moderate accuracy only because most foramina were normal or mild; it missed most severe stenosis and systematically undergraded. In its current form this approach is unsuitable for screening, triage, operative decision-making, or independent report generation.

## Introduction

Lumbar neuroforaminal stenosis is a common degenerative condition, affecting an estimated 103 million people worldwide, with prevalence rising steeply with age.^1^ It involves compression of the exiting nerve root within the intervertebral foramen, and is distinct from central canal stenosis, which affects the cauda equina, and from lateral recess stenosis, which compromises the traversing nerve root.^1^ For spine surgeons, the key safety question for any artificial intelligence (AI) tool that reads these scans is not whether it can correctly label the many normal or mildly narrowed foramina, but whether it reliably identifies severe foraminal narrowing, the finding that corresponds to radiculopathy and influences referral, injections, decompression planning, and fusion discussions.

Two kinds of AI now read spine images. The first is the dedicated deep-learning algorithm: a program trained on thousands of labeled spine MRIs to perform one task, which detects foraminal and lateral recess stenosis with sensitivities approaching 98%.^2-4^ The second is the multimodal vision frontier model (M-VFM), such as Gemini 3 Pro (Google DeepMind, London, UK). These are large, general-purpose AI models trained on vast amounts of internet text, images, and video; they can look at a medical image and answer questions about it in plain language.^5-10^ They are attractive because a clinician can simply ask them to grade a scan, with no stenosis-specific model to build or train. They have not, however, learned the task from labeled foraminal examples in the way a dedicated algorithm has. For any AI tool to be useful in radiology, acceptable overall accuracy is not enough: it must also show adequate sensitivity for the severe findings that require timely intervention.^1,10-12^

Testing such a model on MRI requires one practical step. General-purpose models cannot read scans in the standard hospital imaging format (Digital Imaging and Communications in Medicine, DICOM). We therefore converted each study’s sagittal and axial T2-weighted series into a short video that scrolls through the slices. In a written prompt, we then asked the model to grade each foramen using the same criteria the dataset’s radiologists used. The video preserves what a reader sees on the screen but discards technical information such as slice spacing, acquisition parameters, and exact signal intensities. The primary objective of this study was to measure how closely Gemini 3 Pro’s grades agreed with expert radiologists’ grades of lumbar neuroforaminal stenosis, with particular attention to severe disease. Secondary objectives were overall accuracy, systematic bias in the direction of errors, variation across vertebral levels, and whether a vertebral-level localizer overlay improved performance.

## Methods

### Study Design

We conducted a retrospective diagnostic accuracy study comparing Gemini 3 Pro’s severity grades for lumbar neuroforaminal stenosis with expert radiologist reference grades from the 2024 Radiological Society of North America (RSNA) Lumbar Spine Degenerative Classification Dataset (LSDD). The dataset comprises more than 2,600 lumbar MRI examinations (over 8,500 series: sagittal T2, axial T2, and sagittal T1) contributed by 12 institutions worldwide. Approximately 60 volunteer neuroradiologists and musculoskeletal radiologists, recruited by the RSNA, the American Society of Spine Radiology, and the American Society of Neuroradiology, graded the location and severity of five degenerative conditions at the five lumbar disc levels (L1-L2 through L5-S1). It was assembled in 2024 for the RSNA Lumbar Spine Degenerative Classification AI Challenge hosted on the Kaggle platform^13^ and is the largest publicly available collection of its kind. According to the dataset’s data-resource publication, each annotator graded a single anatomical location and each case was annotated once, with additional reads to establish two-reader agreement obtained only for the challenge test sets^14^; the released label files provide one grade per level and side without reader-level information. The labels used here are therefore single expert annotations rather than formally adjudicated multi-reader consensus. Each examination contributed 10 foraminal-level observations (five lumbar levels, both sides), giving 1,000 foraminal assessments. Neuroforaminal stenosis was graded on the RSNA three-level ordinal scale: normal/mild, moderate, or severe.

The primary analysis focused on the Gemini 3 Pro model operating without localizer assistance (Video 1). A secondary head-to-head comparison evaluated performance when a vertebral-level localizer workflow was incorporated (Video 2).

### Outcome Measures

The primary endpoint was agreement between the model’s grades and the expert reference grades, measured with linearly weighted Cohen’s kappa (κw). Cohen’s kappa expresses agreement beyond what would occur by chance alone (0 indicates chance-level agreement and 1 perfect agreement). The linearly weighted version gives partial credit when the model is one grade away from the reference and none when it is two grades away. The principal clinical safety measure was sensitivity for severe stenosis (the proportion of radiologist-confirmed severe foramina that the model also called severe), because a severe foramen labeled normal or mild is the error most likely to cause harm. Secondary measures were overall accuracy, specificity, and positive predictive value (the probability that a foramen the model calls severe is truly severe). We also report positive and negative likelihood ratios, which express how much a “severe” or “not severe” answer from the model should raise or lower a clinician’s suspicion. Ninety-five percent confidence intervals (95% CI) were calculated for key measures.

### Statistical Analysis

Agreement between model and expert grades was tabulated in a three-by-three confusion matrix. κw was computed across all 1,000 foraminal observations with linear weights (1.0, 0.5, and 0.0 for exact, adjacent, and extreme disagreement); unweighted κ was also calculated, and 95% CIs were derived from the asymptotic (Fleiss–Cohen) standard error. Sensitivity, specificity, positive predictive value, and likelihood ratios were calculated for each grade against the other two combined (one-vs-rest); the likelihood ratios reported in the Results and in Table 1 refer to severe stenosis. To examine how well the model separated neighboring grades, we also compared grades in pairs: severe versus moderate, severe versus normal/mild, and moderate versus normal/mild. For each pair we report the differentiation accuracy and the sensitivity for the higher grade. McNemar’s test was used to determine whether disagreements were random or systematically biased toward under- or over-grading, both for each pair and, as an omnibus test of symmetry (Bowker’s extension), across the full three-by-three table. In the workflow comparison, McNemar’s test for paired data compared overall accuracy and severe-stenosis sensitivity between the standard and localizer-assisted workflows. Two-sided p values below 0.05 were considered significant.

**Table 1.** Statistical Reliability Metrics of the Gemini 3 Pro Model (without Localizer)

Statistical Test	Result
Weighted Cohen’s Kappa (κw)	0.20 (95% CI 0.14–0.26)
Unweighted Cohen’s Kappa (κ)	0.15 (95% CI 0.10–0.20)
Positive Likelihood Ratio (LR+)	7.33
Negative Likelihood Ratio (LR-)	0.84
McNemar’s Test	p < 0.001
Direction of Error	Tendency to Undergrade

Assessment of the model’s diagnostic utility using likelihood ratios and evaluation of systematic bias using McNemar’s test. The direction of error indicates the model’s general tendency when disagreements occur (undergrading versus overgrading). Weighted (κw) and unweighted (κ) Cohen’s kappa are chance-corrected agreement statistics computed across all 1,000 foraminal observations (95% CIs from the asymptotic standard error); likelihood ratios refer to severe stenosis versus all other grades (one-vs-rest); McNemar’s test is the omnibus test of symmetry across the full 3×3 table, with pairwise results in Tables 3 and 4.

**Abbreviations:** CI, Confidence Interval; κ, Cohen’s kappa; κw, linearly weighted Cohen’s kappa; LR+, Positive Likelihood Ratio; LR-, Negative Likelihood Ratio.

### Sample Size and Power Considerations

In plain terms, we calculated how many foramina were needed to show convincingly that agreement was at least moderate, allowing for the fact that the ten foramina from one patient are not independent observations. The primary endpoint was agreement between Gemini 3 Pro and the expert radiologist reference grades for three-level ordinal neuroforaminal stenosis severity (normal/mild, moderate, severe), quantified using linearly weighted Cohen’s kappa (κw) with linear weights (1.0, 0.5, 0.0). Power calculations targeted a one-sided hypothesis test of H_0_: κw ≤ 0.40 vs H_1_: κw = 0.60 at α = 0.05 with 80% power, using planning marginal prevalences consistent with the expected case mix (approximately 0.70 normal/mild, 0.24 moderate, 0.06 severe). The asymptotic variance of κw was approximated using a delta-method approach for multinomial cell proportions and evaluated conservatively at κw = 0.40, yielding a requirement of approximately 150 independent observations. Because each MRI contributes multiple foraminal-level observations (m = 10 per exam; five lumbar levels × bilateral foramina), clustering was incorporated via a design effect (DE) = 1 + (m − 1)ρ, assuming within-exam intraclass correlation ρ = 0.25 (sensitivity ρ = 0.10–0.30). This corresponded to approximately 487 total observations (≈49 examinations), with sensitivity bounds of roughly 285–555 observations (≈29–56 examinations) across the intraclass correlation coefficient (ICC) range.

### MRI Inputs and Series Identification

For each examination, sagittal and axial T2-weighted DICOM series were used. A metadata table containing the dataset was parsed with Python to associate each study ID with the relevant sagittal and axial T2 series IDs, using the series description fields. Only studies featuring both sequences were included in the video generation.

### Video Generation, Model Querying, and Structured Prompting

For each examination, the sagittal and axial T2-weighted series were converted into a single side-by-side video in which both planes scroll together from cranial to caudal (step-by-step details in Supplement A). Each video was uploaded to Gemini 3 Pro through the model’s programming interface by a single script, together with a standardized written prompt (Supplement B). The prompt asked the model to act as a neuroradiologist and review the sagittal and axial images. It then had to assign a grade of normal/mild, moderate, or severe to the left and right foramen at each of the five disc levels (L1-2 through L5-S1), using the RSNA grading criteria. The model was used exactly as released: no training, additional task-specific training (fine-tuning), or other modification of the model was performed, and it was shown no labeled examples from the dataset as part of this study. To allow automated scoring, the model was required to return its grades in a fixed, machine-readable format listing each level, side, and grade. Uploaded files were deleted after each response, and the script recorded a failure whenever a request did not complete or a response did not follow the required format.

### Software and Reproducibility

All preprocessing, submission of videos and prompts to the model, output parsing, statistical analyses, and figure generation were performed using prespecified scripts. Python (v3.14.4) was used for image processing and for submitting videos and prompts to the model, and R (v4.6.0) in RStudio was used for statistical analyses and figure generation. Detailed video-generation parameters are provided in Supplement A; processing parameters were fixed a priori and applied uniformly across examinations for reproducibility.

## Results

The analysis included 1,000 foraminal observations from 100 MRIs, exceeding the a priori requirement of approximately 487 observations (≈49 examinations under the clustering assumptions). Under those assumptions, the sample provided approximately 97% power to detect κw of 0.60 against the minimum acceptable 0.40. Supplement C summarizes the overall results. Agreement between the model and the expert grades was poor: linearly weighted κw was 0.20 (95% CI 0.14–0.26) and unweighted κ was 0.15 (95% CI 0.10–0.20). The confusion matrix (Figure 1) shows where the disagreements lay. Of 704 normal/mild foramina, 672 were graded correctly. Of 240 moderate foramina, only 24 were graded correctly; 201 were downgraded to normal/mild and 15 were upgraded to severe. Of 56 severe foramina, only 10 were graded correctly; 13 were downgraded to moderate and 33 to normal/mild. Upgrading was much less common: 24 normal/mild foramina were called moderate and 8 were called severe, in addition to the 15 moderate foramina called severe. In other words, when the model disagreed with the radiologists, it usually called the foramen less narrowed than they did (247 downgrades versus 47 upgrades).

**Figure 1.**
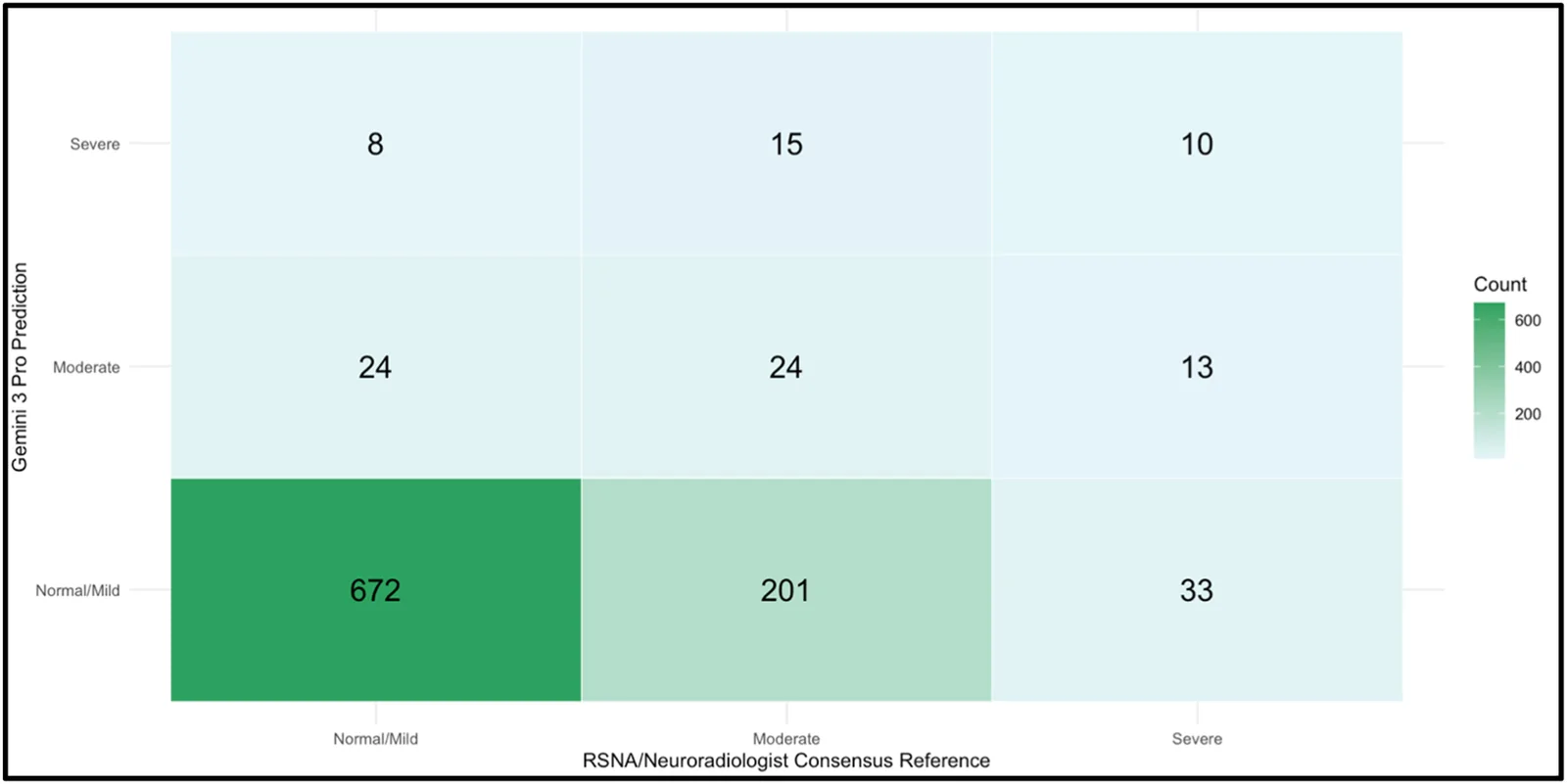
Confusion Matrix Comparing Gemini 3 Pro Classifications (without Localizer) against Expert Radiologist Reference Grades. Heatmap illustrating agreement and disagreement between Gemini 3 Pro predictions (rows/y-axis) and RSNA expert radiologist reference grades (columns/x-axis) for 1,000 foraminal-level observations. Cell annotations indicate case counts. Green cells along the diagonal represent correct predictions, with darker intensities indicating higher case counts. Off-diagonal cells represent misclassifications. Abbreviations: RSNA, Radiological Society of North America

Overall accuracy without the localizer was 70.6% (95% CI, 67.7–73.4%) (Table 2). Because normal/mild foramina made up 70.4% of the dataset, this figure says little about the model’s ability to find disease. Sensitivity for severe neuroforaminal stenosis was 17.9% (95% CI, 8.9–30.4%): of 56 radiologist-confirmed severe foramina, 33 (58.9%; 95% CI, 45.0–71.9%) were called normal/mild and 46 (82.1%; 95% CI, 69.6–91.1%) were called something other than severe (Figure 2).

**Table 2.** Clinical Safety and Accuracy Metrics of the Gemini 3 Pro Model (without Localizer)

Clinical Metric	Observed Result	95% CI
Sensitivity (Severe Cases)	17.9%	8.9–30.4%
Severe Downgraded to Normal/Mild	58.9%	45.0–71.9%
Severe Classified as Non-Severe	82.1%	69.6–91.1%
Overall Accuracy	70.6%	67.7–73.4%

Summary of key clinical performance indicators, focusing on severe-case sensitivity, severe-to-normal/mild critical downgrading, severe cases classified as any non-severe category, and overall model accuracy. Data are presented with 95% confidence intervals (95% CIs).

**Abbreviations:** CI, Confidence Interval.

**Figure 2.**
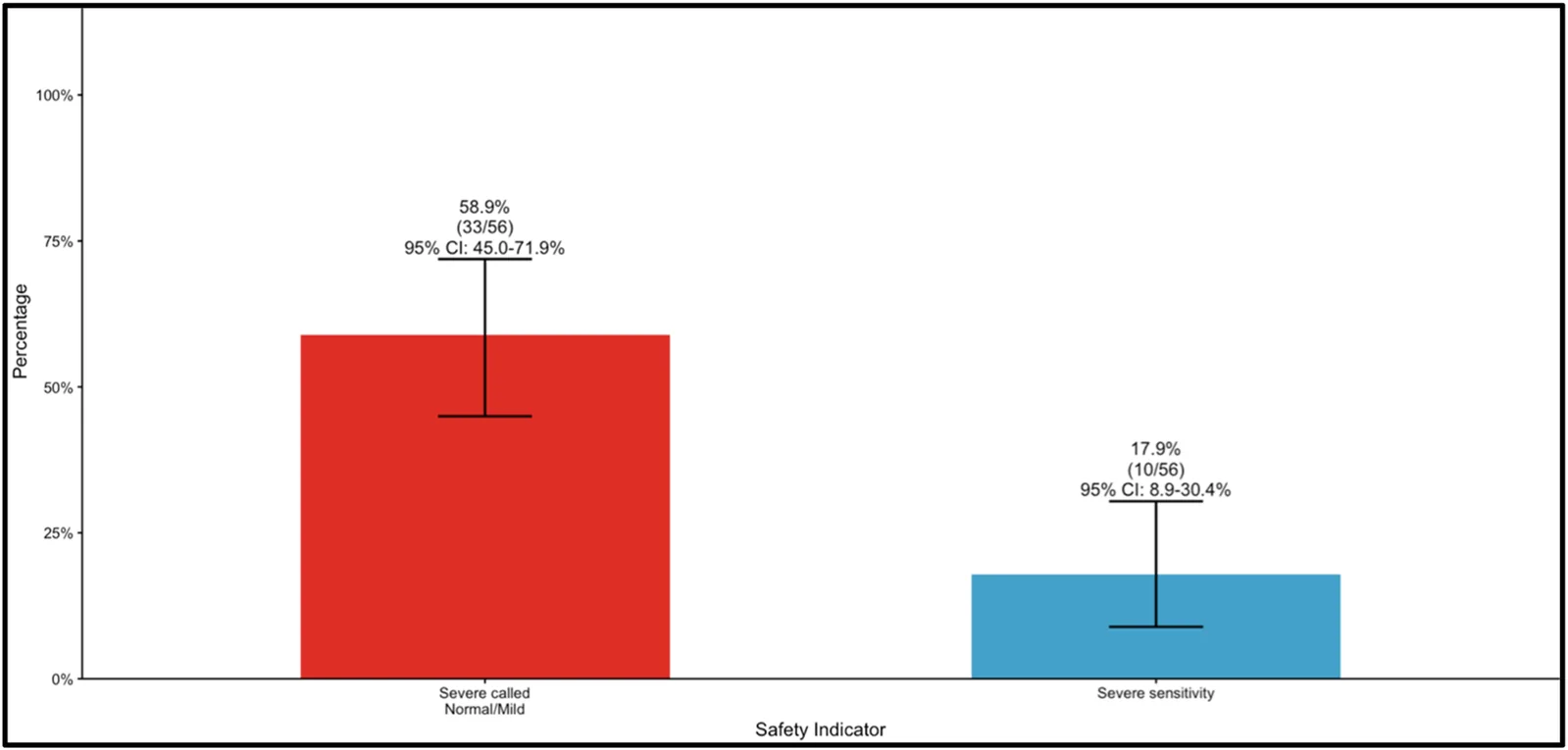
Severe-Case Safety Profile: Sensitivity and Critical Downgrading for Severe Neuroforaminal Stenosis (Gemini 3 Pro without Localizer). Bar chart displaying sensitivity for severe neuroforaminal stenosis and the critical downgrade rate for severe cases classified as normal/mild. Error bars and labels show exact 95% confidence intervals and case counts. Abbreviations: CI, Confidence Interval

Table 1 summarizes what a model output means in practice. A “severe” call raised the odds of true severe stenosis about seven-fold (positive likelihood ratio 7.33), but a “not severe” call barely lowered them (negative likelihood ratio 0.84), so a non-severe output does not reliably exclude severe disease. McNemar’s test confirmed that the disagreements were systematic rather than random (p < 0.001), with undergrading the dominant direction. Grade by grade (Figure 3), the model had high sensitivity for normal/mild foramina (0.95) but low specificity (0.21), reflecting its tendency to assign that label. For moderate stenosis, sensitivity was 0.10, specificity 0.95, and positive predictive value 0.39; for severe stenosis, sensitivity was 0.18, specificity 0.98, and positive predictive value 0.30. Even when the model did call a foramen severe, it was therefore correct less than one time in three.

**Figure 3.**
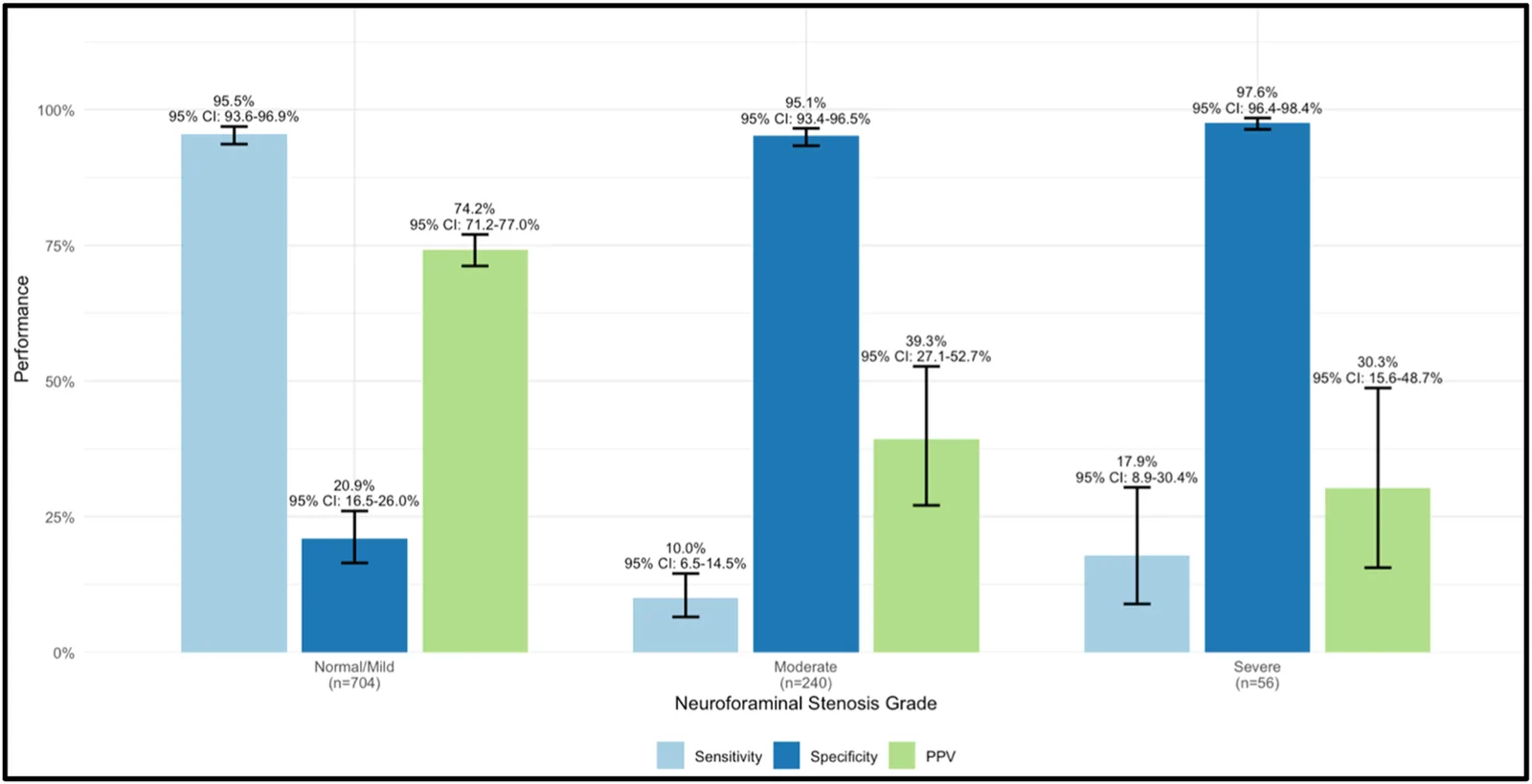
Diagnostic Performance Metrics Stratified by Neuroforaminal Stenosis Severity Grade (Gemini 3 Pro without Localizer). Grouped bar chart showing one-vs-rest sensitivity, specificity, and positive predictive value for each stenosis grade, with reference class counts (normal/mild n = 704; moderate n = 240; severe n = 56) and 95% confidence intervals annotated. Abbreviations: CI, Confidence Interval; PPV, Positive Predictive Value

When the comparison was restricted to pairs of grades (Table 3), the model separated severe from normal/mild foramina with 89.7% accuracy (95% CI, 87.4–91.8), but this largely reflected its habit of calling foramina normal/mild, since severe sensitivity remained 17.9%. It separated severe from moderate foramina with only 11.5% accuracy (95% CI, 8.1–15.7), and moderate from normal/mild with 73.7% accuracy (95% CI, 70.8–76.5) and a sensitivity of 10.0% (95% CI, 6.5–14.5) for the higher grade. Pairwise bias testing (Table 4) showed systematic undergrading for severe versus normal/mild (p < 0.001) and moderate versus normal/mild (p < 0.001), whereas errors between severe and moderate showed no directional bias (p = 0.850).

**Table 3.** Pairwise Diagnostic Accuracy of the Gemini 3 Pro Model (without Localizer)

Comparison Pair	Differentiation Accuracy (95% CI)	Sensitivity for Higher Grade (95% CI)
Severe vs. Moderate	11.5% (8.1–15.7)	17.9% (8.9–30.4)
Severe vs. Normal/Mild	89.7% (87.4–91.8)	17.9% (8.9–30.4)
Moderate vs. Normal/Mild	73.7% (70.8–76.5)	10.0% (6.5–14.5)

Detailed breakdown of the model’s accuracy in distinguishing between specific pairs of severity grades. This table highlights diagnostic uncertainty, particularly in distinguishing severe from moderate neuroforaminal stenosis.

**Abbreviations:** CI, Confidence Interval.

**Table 4.** Pairwise Bias Analysis and Direction of Diagnostic Errors of the Gemini 3 Pro Model (without Localizer)

Comparison Pair	Direction of Error	McNemar P-Value	Interpretation
Severe vs. Moderate	No significant bias	0.850	Random Errors
Severe vs. Normal/Mild	Tendency to Undergrade	< 0.001	Significant Bias
Moderate vs. Normal/Mild	Tendency to Undergrade	< 0.001	Significant Bias

Analysis of systematic errors for specific diagnostic pairs. McNemar’s test determines whether discordant classifications are random or biased. The direction of error indicates whether the model tends to underestimate or overestimate severity in discordant cases.

Accuracy varied by level (Figure 4): 94.0% at L1–L2, 84.5% at L2–L3, 70.0% at L3–L4, 48.0% at L4–L5, and 56.5% at L5–S1. Adding the vertebral-level localizer (Table 5) did not change overall accuracy (70.0% [95% CI, 67.1–72.8%] versus 70.6% [95% CI, 67.7–73.4%]; p = 0.590) but reduced severe sensitivity from 17.9% (95% CI, 8.9–30.4%) to 3.6% (95% CI, 0.4–12.3%; p = 0.043).

**Figure 4.**
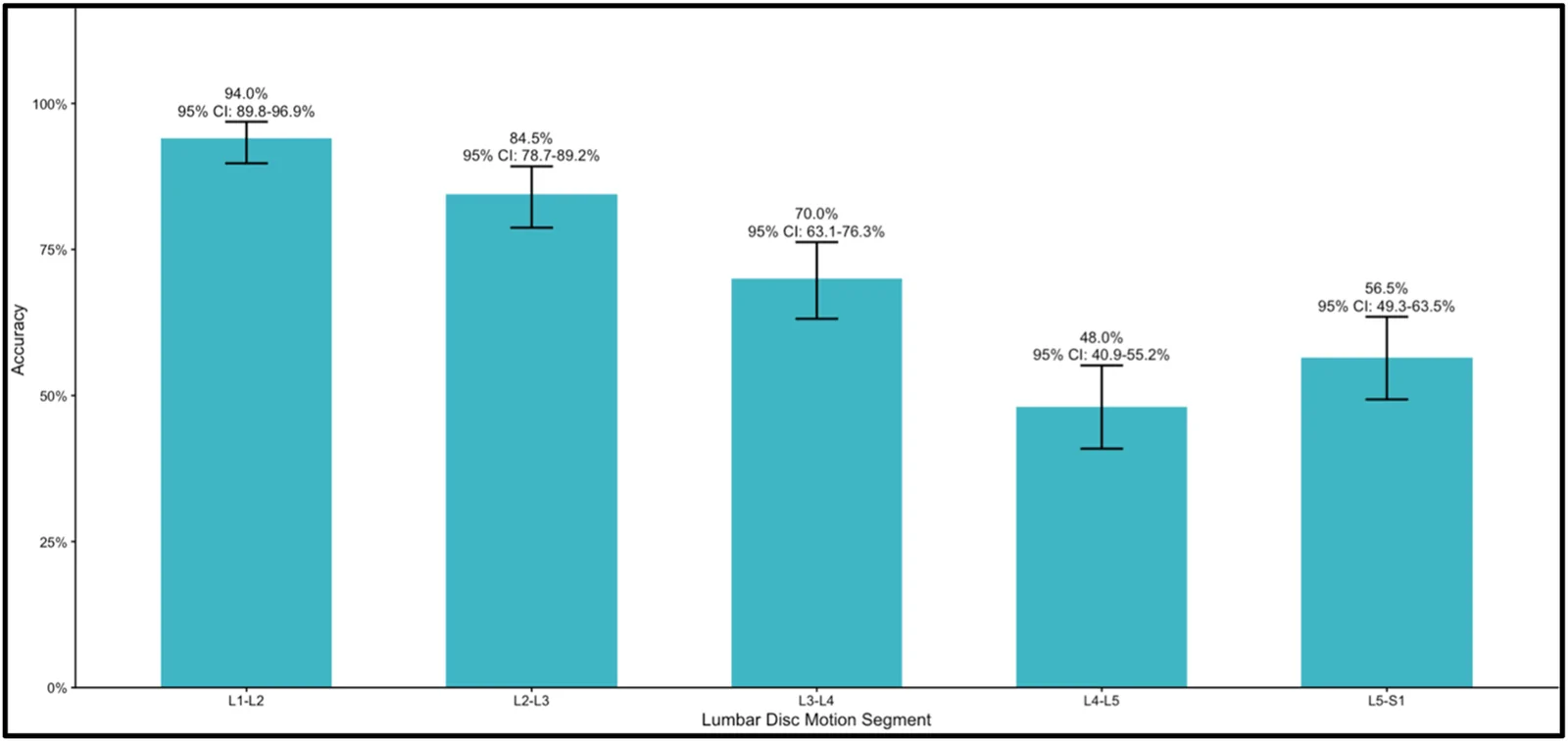
Anatomical Distribution of Diagnostic Accuracy by Lumbar Foraminal Level (Gemini 3 Pro without Localizer). Bar chart displaying diagnostic accuracy for each lumbar disc motion segment (L1-L2 through L5-S1; n = 200 foramina per level). Error bars and labels denote 95% confidence intervals and observed accuracy. Abbreviations: CI, Confidence Interval; L, Lumbar; S, Sacral

**Table 5.** Head-to-Head Comparison of Standard vs. Localizer-Assisted Gemini 3 Pro Workflows

Metric	Gemini 3 Pro	Gemini 3 Pro (with Localizer)	McNemar P-Value
Overall Accuracy (95% CI)	70.6% (67.7–73.4)	70.0% (67.1–72.8)	0.590
Sensitivity for Severe (95% CI)	17.9% (8.9–30.4)	3.6% (0.4–12.3)	0.043

Comparative analysis of the standard Gemini 3 Pro workflow against a workflow using a vertebral-level localizer overlay. P-values indicate the statistical significance of differences in overall accuracy and sensitivity for severe neuroforaminal stenosis between workflows.

**Abbreviations:** CI, Confidence Interval.

## Discussion

In this study, a general-purpose multimodal AI model, used without any task-specific training, graded 1,000 lumbar foramina from videos of their MRI studies. Its agreement with expert radiologists was poor (κw 0.20), and the apparently reasonable overall accuracy of 70.6% was an artifact of the case mix: 70.4% of foramina were normal or mild, and the model called almost everything normal or mild. When disagreements occurred, they ran almost entirely in one direction, toward less severe grades. The model identified fewer than one in five severe foramina, called more than half of them normal or mild, and separated severe from moderate narrowing correctly in only about one case in nine. A “not severe” output therefore did not meaningfully lower the probability of severe disease (negative likelihood ratio 0.84), and even a “severe” output was wrong more often than it was right (positive predictive value 0.30). For a surgeon, these are the findings that matter, and they point in the opposite direction from the headline accuracy.

### Why a general-purpose model struggles with this task

Several features of the method explain the result. First, the model was used as released: we did not train it. In machine-learning terms, training means adjusting a model’s internal settings on a large set of labeled examples (here, foramina graded by experts) until it learns the task. A trained model is then checked on separate validation and test sets, ideally from other institutions, because performance often falls when images come from a different scanner or population.^15-17^ Fine-tuning, which is additional training of an existing model on task-specific labeled data, is the usual way to fix a specific weakness such as missed severe disease. It carries its own requirements for data governance, bias assessment, and transparent reporting.^18-23^ None of these steps was part of this study. The model was simply asked to grade, so its answers reflect whatever it absorbed about spine MRI during its general training, not any instruction in what severe foraminal narrowing looks like.

Second, the input was a video rather than the original images. Converting the series to video preserves what the eye sees but removes slice spacing, acquisition parameters, and quantitative signal information, and the model cannot window, zoom, or scroll back and forth as a radiologist would. Third, the features that separate moderate from severe foraminal stenosis (the degree of perineural fat obliteration, nerve-root compression, and changes in root morphology) are subtle and require fine visual discrimination that current general-purpose models have not demonstrated. Fourth, the model’s answers may be shaped by a conservative tendency to prefer the most common label, which minimizes false positives at the cost of missing disease. Accuracy was lowest at L4–L5 and L5–S1, the levels with the greatest anatomic variability and the most clinically relevant foraminal disease. This, too, is consistent with a model that has not learned the specific anatomy of the task.

The localizer experiment supports this interpretation. We expected that marking the level under review on the sagittal image would help the model find the right foramen. Instead, the overlay left overall accuracy unchanged and cut severe sensitivity from 17.9% to 3.6%. If the model’s main problem were finding the level, the localizer should have helped. That it did not suggests the limitation lies in judging the degree of narrowing once the level is found; the extra visual element may also have distracted the model. Changes to an AI workflow that seem helpful should be tested rather than assumed.

### What this means for spine surgeons

In practical terms, this approach should not be used in its current form for screening, triage, operative decision-making, or independent report generation. Severe lumbar stenosis carries real therapeutic weight: the probability of surgery exceeds 57% over 7–8 years of follow-up.^1,24^ A tool that calls a truly severe foramen normal or mild can create false reassurance, delay referral, or mislead patients who bring AI-generated impressions to clinic. Regulators expect safety and effectiveness to be demonstrated before clinical adoption,^10,12^ and the Food and Drug Administration’s Software as a Medical Device pathway requires valid scientific evidence that a device performs its intended function without causing harm.^11^ A model that misses four of every five severe foramina does not meet that bar for a task with direct surgical implications. Surgeons evaluating any AI imaging tool should ask for grade-specific sensitivity and the direction of its errors, not overall accuracy alone. A model that labels nearly everything normal will look accurate in any population in which disease is uncommon.

### How this compares with dedicated algorithms and with radiologists

Dedicated deep-learning algorithms trained for this task perform far better. Hallinan et al. reported 84.5% recall for lumbar neuroforaminal stenosis, with dichotomous grading agreement of Gwet κ 0.89 versus 0.94–0.95 for subspecialist radiologists.^2^ Bharadwaj et al. separated normal/mild from moderate/severe foraminal stenosis with an area under the receiver operating characteristic curve (AUROC) of 0.92;^25^ for that same binary distinction, our model achieved a sensitivity of only 20.9% despite a specificity of 95.5%. Tumko et al. reported AUROC values of 0.950 for the presence and 0.890 for the severity of foraminal stenosis, exceeding a radiologist panel.^3^ Comparable results have been reported in the cervical spine (Zhang et al., κ 0.644 and AUC 0.89;^26^ Lee et al., κ 0.80 for foraminal stenosis^27^). General-purpose models have done well in narrower settings: Yilihamu et al. reported 92–94% accuracy (κ 0.88–0.89) when GPT-4 classified single lumbar foraminal images,^28^ and Ziegeler et al. found moderate agreement (κ 0.653) when GPT-4 extracted foraminal stenosis from radiology reports, with disagreement driven mainly by underreporting,^29^ which is the same direction of error we observed. Expert radiologists, by contrast, grade foraminal stenosis with excellent reproducibility (κ 0.87–1.0) using established grading systems that correlate with outcomes and guide treatment.^30,31^ The distance between these benchmarks and our results, above all in separating moderate from severe disease, would have to close before a general-purpose model could be considered for clinical use.^9,10^

## Limitations and future directions

This study has limitations. We evaluated a single model at a single point in time, and the results do not generalize to other models or to later versions. Because each case was annotated once and the released label files contain no reader-level information, we could not compare the model’s agreement with human-only inter-reader agreement. The effects of image quality, patient positioning, and scanner differences were not assessed, and we did not test whether a radiologist working with the model would do better than either alone. The dataset is public, so we cannot exclude that some images were encountered during the model’s general training; if anything, this would bias performance upward. Finally, the study tested one MRI-to-video prompting configuration; other ways of presenting the images or framing the prompt might perform differently, although the localizer experiment shows that plausible changes can also make performance worse.

Improving performance will likely require one or more of the following: training on expert-labeled examples that span the full severity range, with adequate representation of severe disease; model designs better suited to subtle morphologic features; or hybrid systems that pair a general-purpose model with a dedicated stenosis algorithm. Adding clinical context such as symptoms and prior imaging may help, with the caveat that text can bias image assessment. Uncertainty estimates that flag low-confidence cases for human review, and prospective studies comparing AI-assisted with standard radiologist workflows, are the natural next steps. Whatever the approach, evaluation should report grade-specific sensitivity, test for systematic bias, and use expert-assigned reference grades rather than developer-selected datasets,^9,10^ with transparent reporting of limitations and failure modes.^9,19^ Our findings add to broader concerns about the readiness of general-purpose AI for autonomous diagnostic tasks in radiology^8,32^: fluent radiology-style language is not evidence of accurate image interpretation.^6^

## Conclusions

A general-purpose multimodal AI model, used without task-specific training, graded lumbar neuroforaminal stenosis on MRI videos with poor agreement with expert radiologists. Its overall accuracy was moderate only because most foramina were normal or mild; it missed most severe stenosis, systematically undergraded, and could not separate moderate from severe disease, and a vertebral-level localizer made severe-case detection worse. In this configuration the approach is not suitable for screening, triage, operative decision-making, or independent report generation, and its outputs should be treated with caution by surgeons, radiologists, patients, and health systems. The consistency of its errors, namely undergrading at the boundary between moderate and severe disease, identifies a clear target for task-specific training and validation before any clinical use.

## Supplemental Material

Supplemental material - Evaluating Multimodal Vision Frontier Models for Grading Neuroforaminal Stenosis on Lumbar Spine MRIs Supplemental material for Evaluating Multimodal Vision Frontier Models for Grading Neuroforaminal Stenosis on Lumbar Spine MRIs by Ayesha Akbar Waheed, Ahmet Kartal, Sohail R. Daulat, Noel F. Manalil, Chiungwen D. Cheng, Ibrahim Hussain, Roger Härtl, Galal A. Elsayed, Harry Gebhard in Global Spine Journal

## Data Availability

The RSNA 2024 Lumbar Spine Degenerative Classification Dataset is publicly available through RSNA/Kaggle in accordance with the dataset’s terms of use. The Python scripts used for DICOM preprocessing, MP4 montage generation (with and without localizer overlays), and inference orchestration, as well as the R scripts used for statistical analysis, are available from the authors upon reasonable request. Derived montage videos may be shared with qualified investigators, subject to the dataset’s data-use and redistribution requirements.*
