# Supplementary material for: Evaluating Multimodal Vision Frontier Models for Grading Neuroforaminal Stenosis on Lumbar Spine MRIs
Source: Global Spine J. 2026 Nov 11:21925682261508040. Online ahead of print. doi: 10.1177/21925682261508040 (PMC13768253; doi:10.1177/21925682261508040)
Supplement: Supplementary file 1 [file sj-zip-1-gsj-10.1177_21925682261508040.zip › Supplement A.docx]

**Video Generation for Model Input**

A Python preprocessing pipeline was used to convert DICOM stacks into MP4 videos suitable for multimodal inference. For each DICOM series, slices were read using a DICOM parser, pixel arrays were extracted, and intensities were windowed to 8-bit grayscale before resizing. In the standard montage workflow, a fixed window center and width were applied (window center 298; window width 670). Pixel values were clipped to the window range, linearly scaled to 0–255, and converted to an unsigned 8-bit format. Each slice was then resized to 512 × 512 pixels using high-quality interpolation.

Sagittal and axial sequences often differed in slice count, so the montage was temporally synchronized by resampling both stacks to a standard frame count. Specifically, the longer of the two stacks determined the number of frames in the output video. Evenly spaced indices spanning the first to last slice were generated for each stack, and the nearest corresponding slice was selected at each time point. The selected sagittal and axial frames were horizontally concatenated to form a 1024 × 512 side-by-side montage (sagittal on the left, axial on the right) and exported as an MP4 video at five frames per second. This approach ensured that both planes progressed smoothly from cranial to caudal (or dataset-consistent order) throughout the full video duration rather than freezing one plane when its stack ended.

Slice ordering in the standard montage workflow relies on the DICOM Instance-Number field when present, which provides consistent within-series ordering in most cases.

**Video Generation with Localizer Overlay**

A second Python preprocessing pipeline was used to produce side-by-side videos with a dynamic localizer overlay to improve cross-referencing between sagittal and axial planes. In this workflow, DICOM slices were sorted using image geometry rather than file name or instance number alone. For each slice, the physical position along the stack progression axis was computed by projecting the DICOM ImagePositionPatient vector onto the slice-normal direction derived from ImageOrientationPatient. Slices were then sorted by this projected coordinate to ensure anatomically correct cranial-to-caudal progression even when metadata or file ordering was inconsistent.

Windowing in the localizer workflow preferentially used the DICOM WindowCenter and WindowWidth tags when available; when those tags were missing or not usable, default values were applied (window center 300; window width 1500). As in the standard workflow, slices were clipped, scaled to 8-bit grayscale, and resized to 512 × 512 pixels. Frames were then converted to RGB for overlay rendering.

In addition to this workflow, a computed localizer line was drawn on the sagittal image to indicate the position of the current axial slice. For each video frame, a “virtual” axial plane was defined using DICOM slice geometry. Because the video advanced by more than one frame per axial slice, the axial-plane geometry was interpolated between adjacent axial slices. The axial plane position was linearly interpolated between consecutive ImagePositionPatient vectors, and the axial plane normal was interpolated between consecutive slice normals derived from ImageOrientationPatient and renormalized to unit length. Pixel data were not interpolated to avoid blur; only the geometric metadata were interpolated to produce smooth motion of the localizer.

The sagittal plane was modeled from its DICOM geometry (Image Position Patient, Image Orientation Patient, and Pixel Spacing). The intersection of the virtual axial plane with the sagittal image plane was computed by substituting the sagittal plane parameterization into the axial plane equation and solving for the resulting line across the sagittal image width. The computed line endpoints were scaled from the original DICOM matrix size to the 512 × 512-pixel resized frame and overlaid as a thin, high-contrast line on the sagittal panel. The sagittal and axial frames were then concatenated into a 1024 × 512-pixel montage and saved as an MP4 file. To provide smoother scrolling and overlay motion, the localizer videos were generated with four video frames per axial slice (total frames = 4 × number of axial slices) and encoded at 15 frames per second.
