# Supplementary material for: Evaluating Multimodal Vision Frontier Models for Grading Neuroforaminal Stenosis on Lumbar Spine MRIs
Source: Global Spine J. 2026 Nov 11:21925682261508040. Online ahead of print. doi: 10.1177/21925682261508040 (PMC13768253; doi:10.1177/21925682261508040)
Supplement: Supplementary file 1 [file sj-zip-1-gsj-10.1177_21925682261508040.zip › Supplement B - Neuroforaminal Stenosis.docx]

You are a neuroradiologist. Analyze the attached sagittal and axial MRI sequences of the lumbar spine.

Task: Grade neuroforaminal stenosis bilaterally (Left/Right) at: L1-L2, L2-L3, L3-L4, L4-L5, L5-S1.

Use a 4-point reference scale for lumbar neuroforaminal stenosis grading criteria (4-point reference scale).

For grading, primarily assess the foramina on sagittal MRI images (use axial sequences/planes as supportive when needed).

Grade each level/side based on the worst (most severe) appearance at that level.

Grading criteria:

- Grade 0 (Normal):
- No fat effacement with normal keyhole appearance.
- Grade 1 (Mild):
- Some effacement of fat in vertical or transverse direction
- Grade 2 (Moderate):
- Near total or total fat effacement without nerve compression
- Grade 3 (Severe):
- Total fat effacement with nerve root compression

Map to the required 3-class output:

- Normal/Mild = Grades 0–1
- Moderate = Grade 2
- Severe = Grade 3

Output requirements:

For EACH level and side, choose exactly one of: “Normal/Mild”, “Moderate”, or “Severe”.

Return the result as a valid JSON object with the key "findings" using this exact structure (no extra keys, no extra text):

{

"findings": [

{ "level": "L1-L2", "side": "Left", "grade": "Normal/Mild" },

{ "level": "L1-L2", "side": "Right", "grade": "Normal/Mild" },

{ "level": "L2-L3", "side": "Left", "grade": "Normal/Mild" },

{ "level": "L2-L3", "side": "Right", "grade": "Normal/Mild" },

{ "level": "L3-L4", "side": "Left", "grade": "Normal/Mild" },

{ "level": "L3-L4", "side": "Right", "grade": "Normal/Mild" },

{ "level": "L4-L5", "side": "Left", "grade": "Normal/Mild" },

{ "level": "L4-L5", "side": "Right", "grade": "Normal/Mild" },

{ "level": "L5-S1", "side": "Left", "grade": "Normal/Mild" },

{ "level": "L5-S1", "side": "Right", "grade": "Normal/Mild" }

]

}
